# Synthesis and Characterization of Mesoporous Mg- and Sr-Doped Nanoparticles for Moxifloxacin Drug Delivery in Promising Tissue Engineering Applications

**DOI:** 10.3390/ijms22020577

**Published:** 2021-01-08

**Authors:** Georgia K. Pouroutzidou, Liliana Liverani, Anna Theocharidou, Ioannis Tsamesidis, Maria Lazaridou, Evi Christodoulou, Anastasia Beketova, Christina Pappa, Konstantinos S. Triantafyllidis, Antonios D. Anastasiou, Lambrini Papadopoulou, Dimitrios N. Bikiaris, Aldo R. Boccaccini, Eleana Kontonasaki

**Affiliations:** 1 School of Physics, Faculty of Sciences, Aristotle University of Thessaloniki, 54124 Thessaloniki, Greece; gpourout@physics.auth.gr (G.K.P.); itsamesidis@auth.gr (I.T.); 2 Department of Materials Science and Engineering, Institute of Biomaterials, University of Erlangen-Nuremberg, 91058 Erlangen, Germany; liliana.liverani@fau.de (L.L.); aldo.boccaccini@fau.de (A.R.B.); 3 School of Dentistry, Faculty of Health Sciences, Aristotle University of Thessaloniki, 54124 Thessaloniki, Greece; antheo@dent.auth.gr (A.T.); anastasiabeketova@yahoo.com (A.B.); 4 Pharmadev, UMR 152, Université de Toulouse, IRD, UPS, 31400 Toulouse, France; 5 School of Chemistry, Faculty of Sciences, Aristotle University of Thessaloniki, 54124 Thessaloniki, Greece; marlazach@chem.auth.gr (M.L.); evicius@gmail.com (E.C.); x.pappa@yahoo.com (C.P.); ktrianta@chem.auth.gr (K.S.T.); dbic@chem.auth.gr (D.N.B.); 6 Center for Interdisciplinary Research and Innovation (CIRI-AUTH), Balkan Center, 57001 Thessaloniki, Greece; 7 Department of Chemical Engineering and Analytical Science, University of Manchester, Manchester M1 3AL, UK; antonios.anastasiou@manchester.ac.uk; 8 School of Geology, Faculty of Sciences, Aristotle University of Thessaloniki, 54124 Thessaloniki, Greece; lambrini@geo.auth.gr

**Keywords:** mesoporous nanoparticles, drug loading/release, moxifloxacin, human erythrocytes, periodontal ligament cells

## Abstract

Mesoporous silica-based nanoparticles (MSNs) are considered promising drug carriers because of their ordered pore structure, which permits high drug loading and release capacity. The dissolution of Si and Ca from MSNs can trigger osteogenic differentiation of stem cells towards extracellular matrix calcification, while Mg and Sr constitute key elements of bone biology and metabolism. The aim of this study was the synthesis and characterization of sol–gel-derived MSNs co-doped with Ca, Mg and Sr. Their physico-chemical properties were investigated by X-ray diffraction (XRD), scanning electron microscopy with energy dispersive X-ray analysis (SEM/EDX), transmission electron microscopy (TEM), Fourier transform infrared spectroscopy (FTIR), X-ray fluorescence spectroscopy (XRF), Brunauer Emmett Teller and Brunauer Joyner Halenda (BET/BJH), dynamic light scattering (DLS) and ζ-potential measurements. Moxifloxacin loading and release profiles were assessed with high performance liquid chromatography (HPLC) cell viability on human periodontal ligament fibroblasts and their hemolytic activity in contact with human red blood cells (RBCs) at various concentrations were also investigated. Doped MSNs generally retained their textural characteristics, while different compositions affected particle size, hemolytic activity and moxifloxacin loading/release profiles. All co-doped MSNs revealed the formation of hydroxycarbonate apatite on their surface after immersion in simulated body fluid (SBF) and promoted mitochondrial activity and cell proliferation.

## 1. Introduction

Bone repair and regeneration are still challenging issues for orthopedics and maxillofacial surgery. In many cases, the self-healing capacity of bone tissue in large bone defects can be compromised due to trauma or tumor resection, as well as osteoporosis in elderly people or secondary infection. In orthopedics, it is anticipated that 2–5% of all the procedures related to implant placement will present bacterial infections [1] but this number can be increased to 50% when open fracture is involved [2]. Particularly for treatment of infected bone defects, typical procedures suggest the surgical removal of the involved bone fragments in combination with systemic antibiotics administration, and the utilization of bone grafts to regenerate the lost bone structure [3]. The adverse effects of these treatments are often painful extra surgeries with large social costs causing systematic toxicity and resistance to antibiotics. All the above underline the need for the development of novel materials that can promote bone regeneration while at the same time being able to administrate antibiotic substances locally to prevent bacterial infection. 

Different carrier and delivery systems for local release of antibiotics to control infection and assist in bone regeneration are continuously developed [4]. Ιn this field, silica-based mesoporous nanoparticles (MSNs) have been proposed as promising drug vehicles [5]. They are produced using sol–gel-based methods with the addition of cationic surfactants as templating agents to form nanosized ordered structures with pores of 2 to 50 nm [6]. They posses an active surface rich in silanols, capable of binding to a variety of functional molecules, extending their utility to different biomedical applications [7]. They present large surface area and controlled porosity that allows fine control of the release kinetics of the uploaded drugs. Therefore, in comparison to other delivery systems described in the literature, they show better loading ability and more sustained and prolonged drug release attributed to silanol-containing surface, making them suitable for local delivery of antibiotics and osteoinductive and angiogenic growth factors [7]. However, despite their ability to deliver different cargos, they are non-bioactive materials, without apatite forming ability and osteogenic or angiogenic properties to be used in bone filling applications. The most widely applied materials for bone regeneration are bioactive glasses that have been extensively used in regenerative medicine due to their excellent bioactivity, osteostimulating properties and degradability [8]. There is a large variety of forms and compositions mainly in the systems SiO_2_-CaO or SiO_2_-CaO-P_2_O_5_, also commercially available, advocated for regeneration of bone, as well as dental cementum and dentin [5,6,9,10]. These bioactive materials are capable of inducing in vitro osteogenesis and in vivo bone formation through the release of calcium, magnesium and silicon ions, which compel their use for bone scaffolds fabrication [7,11]. 

Bioactive calcium-silicate mesoporous nanoparticles have been developed by the incorporation of calcium ions in the glass network of silica [12,13]. These mesoporous nanoparticles are more reactive, as they demonstrate more rapid ionic release and mineral precipitation on their surface in biological fluids, as compared to conventional non-porous materials [14]. 

Various metallic ions such as Mg, Ag, Sr, Cu, Zn etc. [15,16,17,18] can be incorporated to MSNs, that could further exploit their unique properties. The incorporation of metallic elements in the siliceous framework of mesoporous bioactive glasses (MBGs) through the sol–gel process takes place by the addition of metallic ion salts as precursors in the sol. However, this process affects the condensation stage, disrupts the silica network by creating deficiencies and may negatively influence the mesoporous structure of the nanoparticles, their homogenous size, pore volume, surface area and the amount of available silanols that are essential for effective loading of molecules, restricting their overall efficacy [12,15,16,17,18,19,20]. This is further complicated by the simultaneous incorporation of multiple elements, where synergistic or antagonistic mechanisms exist on the preferable ion incorporation into the silica network [18,20,21]. 

The synthesis of multiple ion co-substituted mesoporous bioactive nanoparticles with high surface area is demanding and only limited literature exists in the field, although the complementary therapeutic effect of different ions could be beneficial in tissue regeneration applications. For example, magnesium plays a significant role in bone metabolism [22,23] and half of the total amount of magnesium in the human body is located in bones. So far, it has been reported that magnesium-containing bioactive glasses present bioactive behavior, controlled rates of biodegradation, increased hardness and can stimulate osteoblast proliferation and differentiation [24]. Similarly, strontium is another important trace element in the human body, which plays a dual role in bone metabolism by stimulating bone formation and inhibiting bone resorption. Some oral drugs containing Sr, like strontium ranelate, have been clinically used for minimizing the risk of osteoporotic bone fractures [25]. A recent study revealed that Sr-incorporated MBG scaffolds promoted regeneration of osteopenic bone defects in mice [25]. Therefore, the simultaneous introduction of Mg and Sr into calcium silicate mesoporous bioactive glass nanoparticles (MSNs) could promote synergetic osteostimulating effects especially in cases of reduced bone quality due to osteoporosis. Limited research exists on multiple ion incorporation of MSNs and the novelty of the present work is represented by the development of MSNs co-doped with Ca, Mg and Sr ions.

Besides incorporating elements with potential regenerating ability, mesoporous nanoparticles are advantageous in bone infections as they can simultaneously administrate locally antibiotics for bacterial elimination. Bone infections are usually hard to treat, while in order to achieve a therapeutic effect, high parenteral doses of antibiotics are required [26]. Systemic administration of antibiotics has been correlated to ineffective amount at the target site, hypersensitivity reactions, nephrotoxicity, gastrointestinal intolerance, bacterial resistance and allergic reactions and interaction with other medications. Thus, current treatment protocols use strategies that eliminate systemic administration and promote the local delivery of drugs/antibiotics to control and eliminate bone and periodontal tissues infections [27,28]. For effective control of bone infection antibiotics that can penetrate to bone tissue such as tetracyclines, cephalosporins, clindamycin, carbapenems, vancomicine, and fluoroquinolones are usually administrated [29]. Furthermore, local delivery systems based on mesoporous bioactive glasses have been developed for gentamicin recently [30]. However, due to the problems of bacterial resistance, alternatives for the currently used antibiotics are needed. Moxifloxacin (MOX) is one such alternative antibiotic. It belongs to the fourth-generation of fluoroquinolone antibiotics and presents antimicrobial activity against a broad spectrum of aerobic and anaerobic bacteria, including S. aureus, which is the main bacterial pathogen of osteomyelitis [31]. Moxifloxacin is superior as compared to ciprofloxacin, levofloxacin and ofloxacin against Staphylococcus aureus [32], and therefore could be applied locally for bacterial elimination. 

In this framework, the purpose of the present study was to synthesize Sr and Mg containing calcium silicate mesoporous nanoparticles by the sol–gel method for delivery of the antibacterial drug (moxifloxacin) and to investigate their structure, properties and drug loading/release profiles.

## 2. Results

### 2.1. Fourier Transform Infrared Spectroscopy (FTIR)

**Figure 1:**
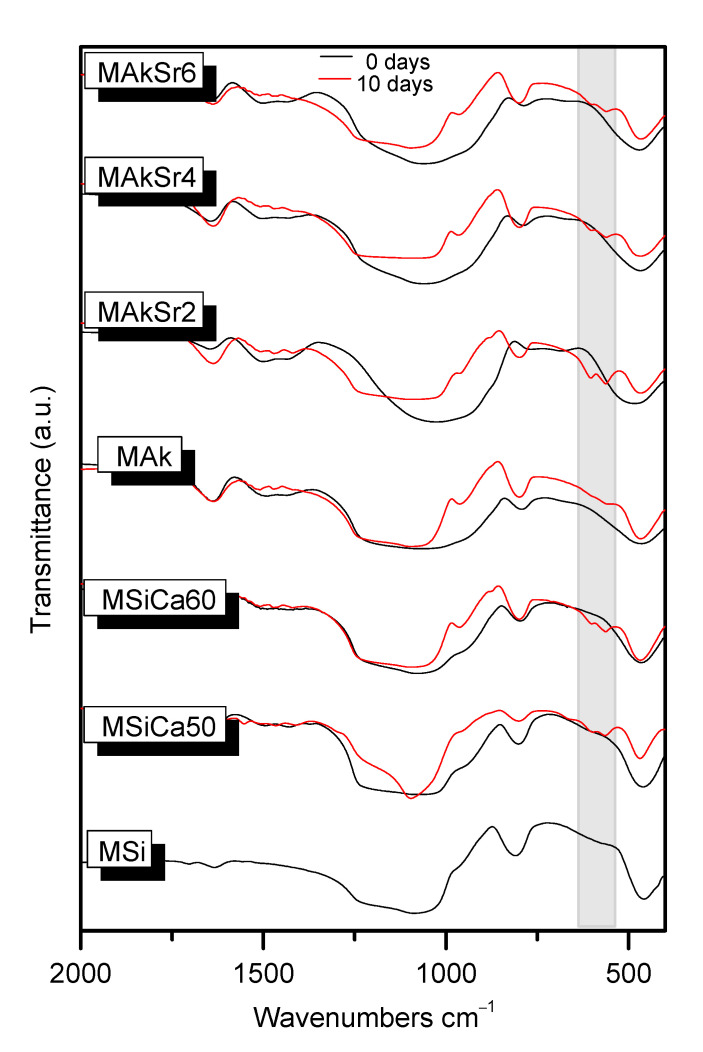
FTIR spectra of all mesoporous silica-based nanoparticles (MSNs) before (0 days) and after 10 days of immersion in simulated body fluid (SBF).

The FTIR spectra of all MSNs presented the characteristic bands of amorphous silicate glasses (Figure 1). Specifically, the peak at 470 cm^−1^ is attributed to the vibration of the Si–O–Si bending mode, the band at 800 cm^−1^ to the symmetric stretching vibration of Si–O. Moreover, there is a shoulder at approximately 960 cm^−1^ which is correlated to the stretching vibration of Si–OH bonds [33,34,35]. The peak located at around 1010 cm^−1^ corresponds to the Si–O–Si asymmetric stretching mode (Q2). Additionally, the peak around 1640  cm^−1^ can be assigned to absorbed water, C-O stretching vibrations are observed in the range of 1405–1505  cm^−1^ and the peak in the range of 750–850  cm^−1^ corresponds to the Si–O–Si stretching vibration (Q0) [19,20,36,37,38]. Additionally, a lower frequency shoulder around 690 and 660 cm^−1^ is ascribed to the Si-O-Mg [39] and Si–O–Ca vibration modes [40], respectively.

### 2.2. X-ray Diffraction (XRD)

**Figure 2:**
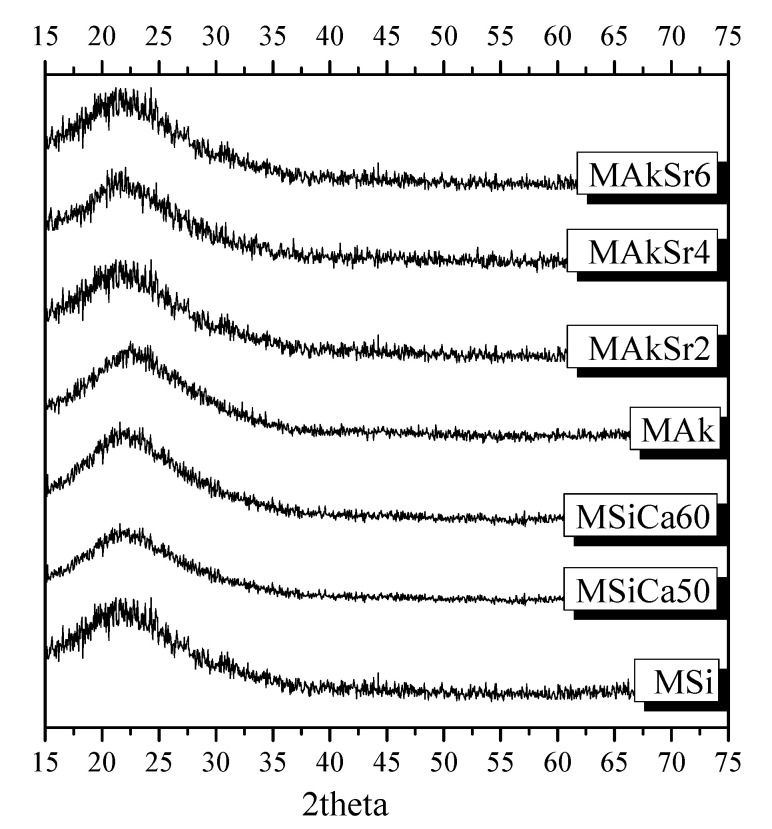
XRD diffractograms of the synthesized mesoporous silica-based nanoparticles (MSNs).

XRD patterns of all MSNs revealed the presence of 100% amorphous glasses (Figure 2).

### 2.3. Particle Size Distribution and Z-potential Measurements by Laser Dynamic Light Scattering (DLS)

**Figure 3:**
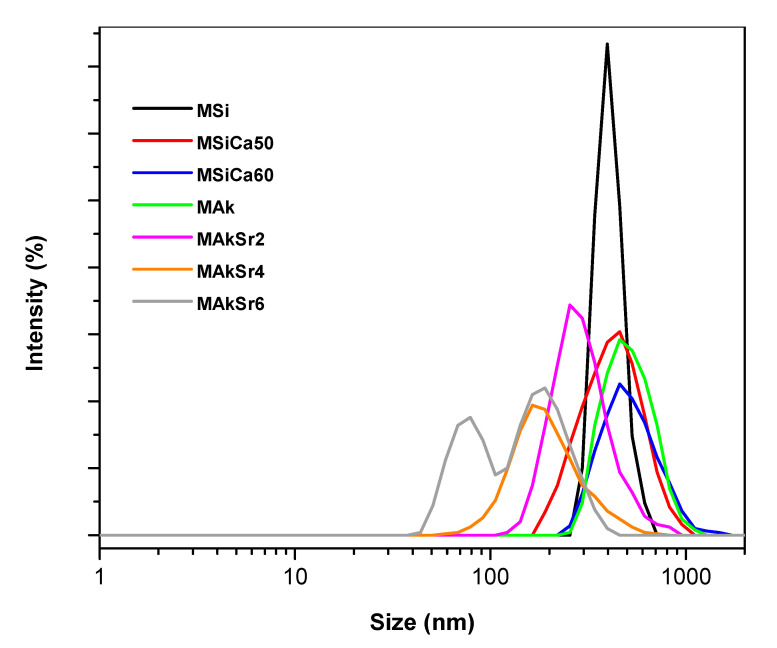
Size distribution of mesoporous silica-based nanoparticles (MSNs) measured by dynamic light scattering (DLS).

The particle size distribution of the MSNs is shown in Table 1 and Figure 3. As can be seen, the particles sizes range from 151.9 to 534.7 nm. MAkSr6 presented two different populations with average size 78.8 and 190 nm, respectively. All MSNs presented negative ζ-potential ranging from −26.7 ± 0.4 to −15.7 ± 0.4.

### 2.4. Brunauer–Emmett–Teller (BET) and Brunauer–Joyner–Halenda (BJH)

**Figure 4:**
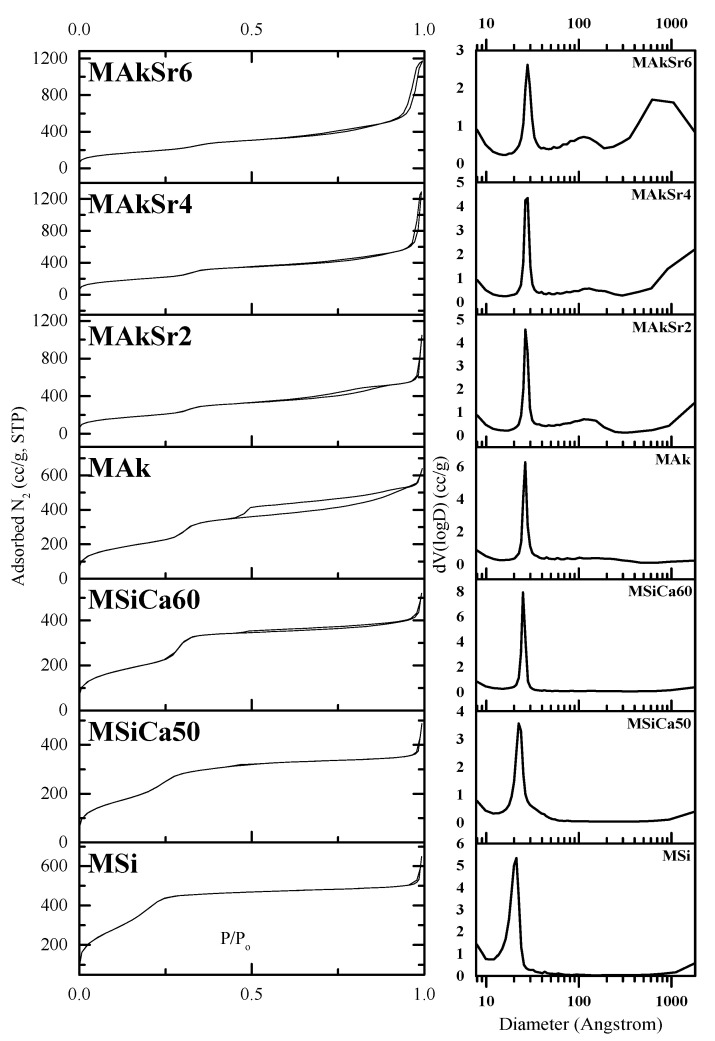
Nitrogen adsorption–desorption isotherms (**left**) of MSNs and their associated pore size distribution (**right**).

The N2 adsorption step of all samples’ isotherms exhibit an IV(b) type, according to the updated IUPAC classification (Figure 4) The IV(b) type, which is a completely reversible isotherm, is usually observed for materials with smaller mesopores; type IV(b) isotherms could be attributed to conical and cylindrical mesopores closed at the tapered end [41]. The desorption step (hysteresis loop) is between H2(a) and H4 for MAk but only at higher P/Po, i.e., corresponding to relatively larger pores, ca. > 3 nm with various distributions of pore types and pore diameters [42]. Almost no hysteresis loop is observed in the desorption isotherms of the rest of the samples. The desorption isotherms present the typical characteristics of mesoporous materials with uniform mesopores, however they seem to have been affected by the MSNs structural features and are representative of a range of mesoporous materials such as silica gels and porous glasses. An additional feature, observed in all samples, is the steep increase in sorbed N2 at very high P/Po, ca. > 0.95, which can be attributed to inter-particle voids and defects on particles surface that generate high external surface. The smaller and closely packed particles of the Sr-containing samples, especially of MAkSr4 and MAkSr6, shown in the SEM microphotographs of Figure 4, may further support their high textural porosity. The specific surface area of the MSNs was between 1279 and 668 m^2^/g, the pore volume between 1.994 and 0.753 cm^3^/g, and the average pore size between 3.1 and 2.4 nm, as can be seen in Table 1. The pore size distribution of most of the MSNs is narrow (Figure 4), centered at about 3 nm, being typical of MCM-41 type materials with hexagonal ordering of channel-like mesopores with similar size.

### 2.5. Scanning Electron Microscopy (SEM) with Energy Dispersive X-ray Analysis (EDX)

**Figure 5:**
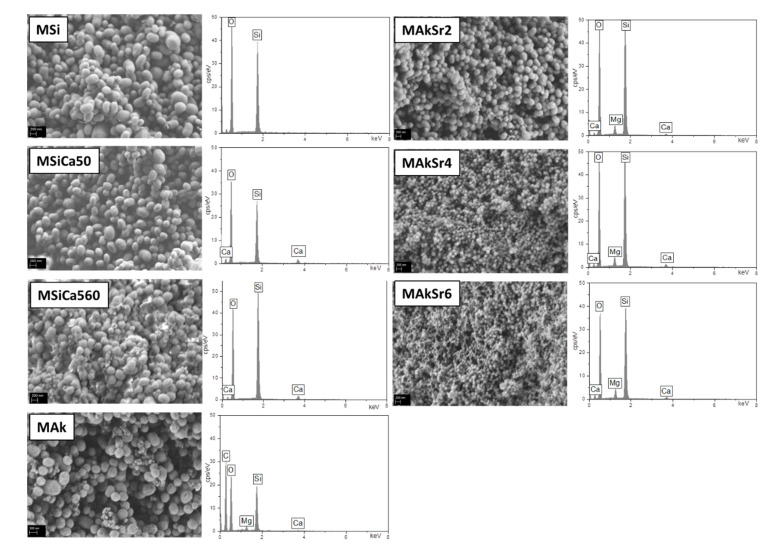
SEM micrographs (45.000× magnification, scale bar for MAk 300 nm, for the rest 200 nm) of the synthesized mesoporous silica-based nanoparticles (MSNs). The respective EDX spectra are incorporated.

The SEM micrographs of the synthesized MSNs are presented in Figure 5. A round or elliptical shape is observed for the MSi, MSiCa50 and MSiCa60 nanoparticles, while small round nanoparticles are observed for the rest of the MSNs. A distinct reduction in size is observed by the incorporation of strontium as shown from the microphotographs of MAkSr2, MAkSr4 and MAkSr6 nanoparticles. As shown from the EDX analysis, magnesium and strontium peaks are present in the respective spectra, suggesting successful incorporation.

### 2.6. Transmission Electron Microscopy (TEM) Analysis

**Figure 6:**
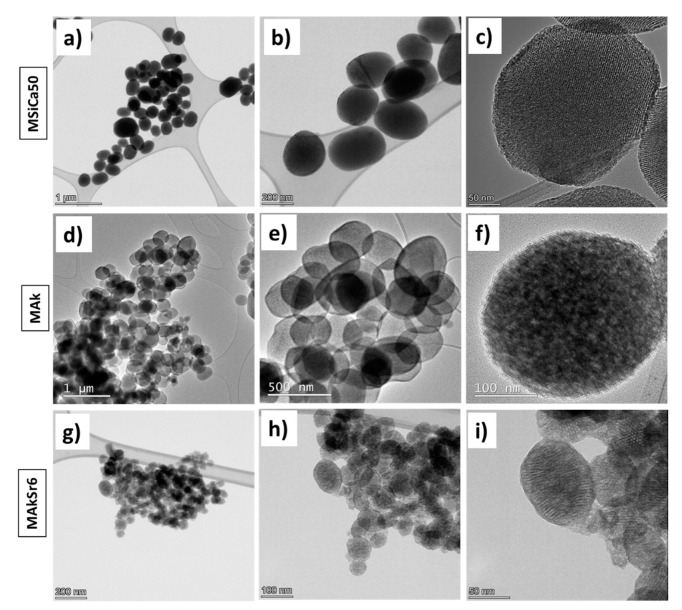
Indicative TEM images: (**a**,**b**) particle size and morphology of the MSiCa50 nanoparticles; (**c**) identification of the hexagonal mesoporous channels in the MSiCa50 nanoparticles (NPs); (**d**,**e**) particle size and morphology of the MAk nanoparticles; (**f**) identification of hexagonal mesoporous structure in MAk NPs; (**g**,**h**) particle size and morphology of the MAkSr6 nanoparticles; (**i**) identification of hexagonal mesoporous structure in MAkSr6 NPs.

Representative TEM images are shown in Figure 6. Round-shaped or slightly elipsoid nanoparticles with mesoporous structure are observed. For MSi, MSiCa50, MSiCa60 and MAk, the size of the nanoparticles (NPs) was in the range between 200 and 320 nm, with homogeneous shape and size, in accordance with SEM micrographs. The addition of Sr resulted in the reduction in the NPs size and this is apparent for the MAkSr4 (average diameter of 130 nm) and MAkSr6 (average diameter of 90 nm). The high magnification TEM images of MSiCa50 and MSiCa60 reveal the presence of hexagonal ordered mesoporous channels. Parallel pore channels are present, which indicates that ion doping (samples MSiCa50 and MSiCa60) has not affected the characteristic mesoporous long period order. Round shaped nanoparticles with rough surface and small void pores that coexist with the mesoporous structure are formed when Mg and Sr are incorporated. The hexagonal mesoporous structure is retained even at the highest amount of strontium incorporation (MAkSr6).

### 2.7. X-ray Fluorescence Spectroscopy (XRF)

The chemical composition of all MSNs as detected by XRF is presented in Table 2. By comparing the nominal and the detected mol% amounts, a limited ion incorporation is observed especially for calcium and magnesium. However, the amount of calcium seems to increase by the incorporation of strontium in the MAkSr2 and MAkSr4 nanoparticles with the exception of MAkSr6, with the highest amount of added Sr.

### 2.8. Apatite Forming Ability in c-SBF

The FTIR spectra of all MSNs after soaking in simulated body fluid (SBF) for 10 days is presented in Figure 1. After soaking for 10 days, the spectra of all MSNs except of MS revealed that hydroxycarbonate apatite (HCAp) was formed on the surface of the specimens. This is verified by the appearance of the two bands at 610–600 and 560–550 cm^−1^ due to the P–O bending vibration and the sharpening and shifting towards lower wavenumbers of the broad peak at 900–1200 cm^−1^ assigned to the bending of the (PO_4_)^3–^ group [43,44,45]. Furthermore, the sharpening of the peak at 780–800 cm^−1^ corresponds to the Si–O–Si stretching vibration which is justified by the polycondensation step of silanols [44,45]. 

**Figure 7:**
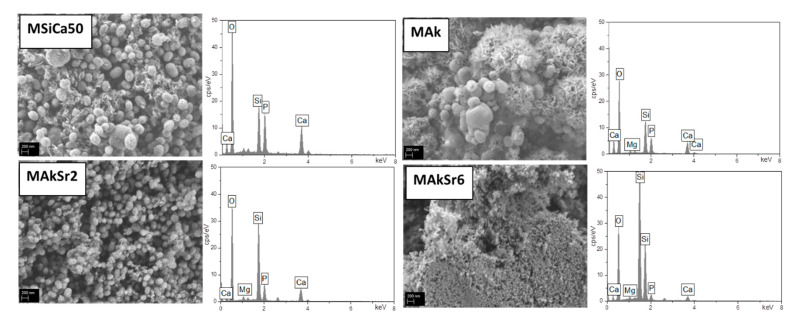
SEM micrographs (45.000× magnification, scale bar 200 nm) with EDX spectra of MSiCa50, MAk, MAkSr2 and MAkSr6 after 10 days in SBF.

Representative SEM micrographs with EDX spectra of MSiCa50, MAk, MAkSr2 and MAkSr6 after 10 days in SBF are presented in Figure 7. Cauliflower-like crystalline apatite is observed on the surface of MSiCa50 and MAk nanoparticles whereas a roughening of the surface of the Sr-doped MSNs suggests the onset of apatite formation, as verified by both EDX and FTIR analyses.

### 2.9. Drug Loading and Release

Table 3 summarizes the results of drug loading determination for each sample. The rates varied from 2 to 38% indicating a low to medium loading capacity. MSiCa50 and MSiCa60 MSNs exhibited higher drug content than that of pure silica (MSi), revealing that the addition of Ca in the system enhances the loading capacity. 

**Figure 8:**
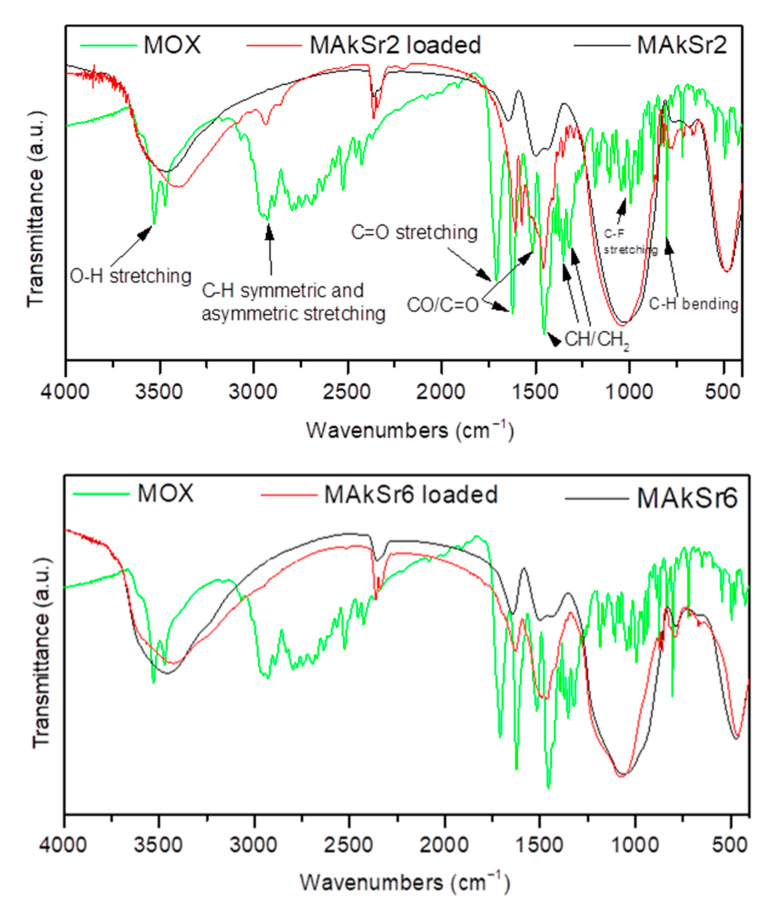
FTIR spectra of MAkSr2 and MAkSr6 before and after loading with moxifloxacin.

In Figure 8, indicative FTIR spectra of the loaded MAkSr2 and MAkSr6 are presented with the spectrum of moxifloxacin as reference, verifying their different amounts of loading. The appearance of charactistic peaks corresponding to moxifloxacin in the spectrum of the loaded MSNs verifies its presence in the nanoparticles. The bands at 1415 to 1475 cm^−1^, 2520 cm^−1^, and 1356 cm^−1^ present in the MOX spectrum are attributed to the vibrations of ((CH)–CH2), ν(-NH_2_^+^) and δ_b_(-CH_2_) respectively [46]. The peak at 1620 cm^−1^ is attributed to the NH bending vibration (presence of quinolones) [47,48,49], the peaks at 802 and 991 cm^−1^ to C-H bending, and the peak around 1710 cm^−1^ to the vibration of δ_b_(COO-) deformation [46]. The absence of the most characteristic peaks of moxifloxacin from the spectrum of MAkSr6 corroborates with the lowest amount of loading for these MSNs (Table 3).

**Figure 9:**
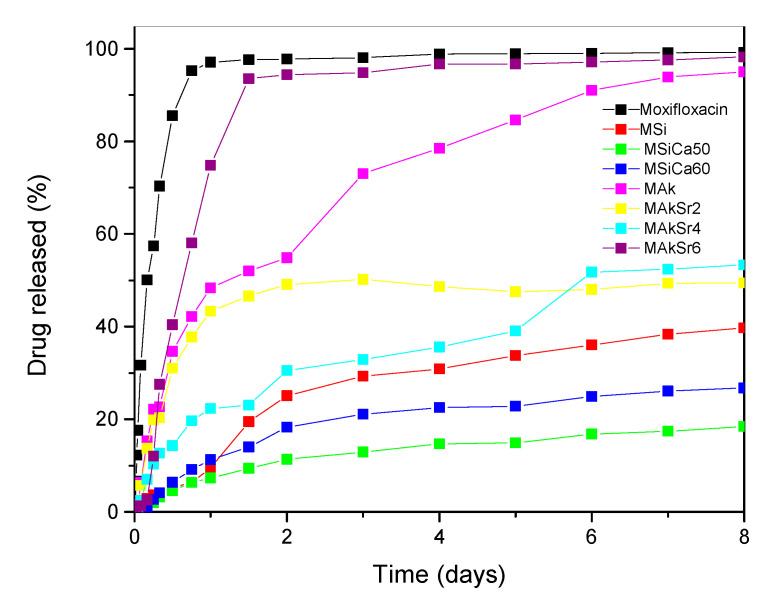
In vitro release rate of moxifloxacin from mesoporous silica-based nanoparticles (MSNs) at pH 7.4.

In Figure 9, dissolution studies for all MOX-loaded MSNs can be observed. Pure moxifloxacin, a highly hydrophilic drug (water solubility: 168 mg/L), is (as expected) rapidly released in the SBF medium and reaches a plateau of 98% in less than 24 h. On the contrary, MSiCa50 nanoparticles exhibited the lowest and most controlled release rate (~18% of MOX was released after 7 days), presumably due to the strong interactions evolved between the MSN system and the drug, while the highest dissolution rate occurred in the case of MAkSr6 sample where a very quick release is also recorded (more than 90% of MOX was released after 1.5 day). In all other cases, the release lasted approximately 7 days, and the rates varied from 26 up to 94%.

### 2.10. In Vitro Biocompatibility Assay

**Figure 10:**
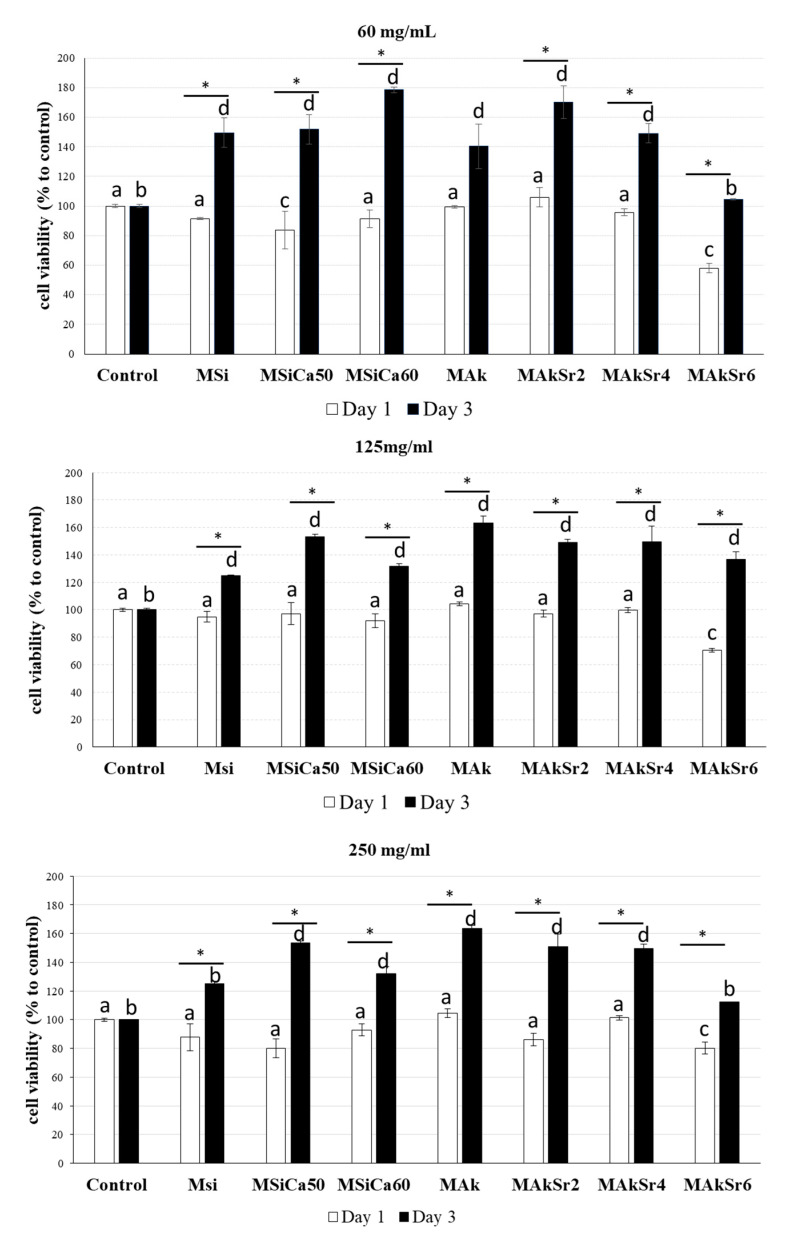
MTT results of cell viability at different concentrations of mesoporous silica-based nanoparticles (MSNs) (μg/mL). * indicates statistically significant difference (*p* < 0.05) between Day 1 and Day 3, while different letter above columns indicates statistically significant differences from control at Day 1 (**a**,**c**) and Day 3 (**b**,**d**).

The MTT analysis revealed that none of the tested groups presented cytotoxicity (Figure 10). In most cases, MSNs eluates promoted mitochondrial activity and cell proliferation. At day 1, a slight decrease in cell proliferation compared to positive control was reported in all tested groups except MAk (all concentrations), MAkSr2 (60 μg/mL) and MAkSr4 (125 and 250 μg/mL). This decrease was statistically significant (*p* < 0.001) for MSiCa50 (250 μg/mL) and MAkSr6 (all concentrations). At day 3, cell proliferation presented a remarkable increase in all tested groups. Cell proliferation increased significantly in all groups (*p* < 0.001) except MSi (125 and 250 μg/mL) and MAkSr6 (60 and 250 μg/mL). Although no dose dependency was clearly observed, the most beneficial effect on cell proliferation was reported in the cases of MAkSr2 and MSiCa60 (60 μg/mL) (*p* < 0.05). Furthermore, a distinct increase in cell proliferation was observed for MAkSr2 and MAkSr4 at all tested concentrations at day 3 (*p* < 0.001). The least increase in cell proliferation was recorded for MAkSr6 that presented negative effects on cell proliferation at day 1 and a positive effect at day 3, being significant at 125 μg/mL.

### 2.11. Hemolysis Assay

**Figure 11:**
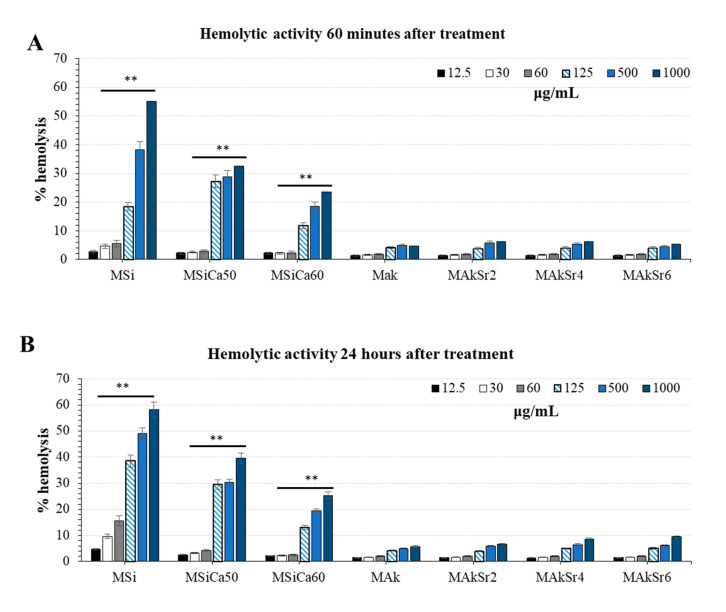
Hemolysis of human red blood cells (RBCs) upon incubation with mesoporous silica-based nanoparticles (MSNs) at different concentrations (12.5, 30, 60, 125, 500 μg/mL) and 37 (**A**) after 60 min and (**B**) after 24 h of incubation. The results are expressed as % of positive control (hemolysis buffer). Data are mean ± SD (n = 10 in each group). ** indicates statistically significant difference (*p* < 0.001).

After the first 60 min of incubation at body temperature, the samples were spun down for the detection of hemoglobin released from hemolyzed red blood cells (RBCs) (Figure 11). Hemolysis of MSi appeared at 30 μg/mL and was improved in the presence of calcium ions (MSiCa60, MSiCa50) in accordance with previous findings [50]. Interestingly, magnesium and strontium-doping of MSNs (MAk, MAkSr2, MAkSr4 and MAkSr6) further improved hemocompatibility. A slight hemolysis was observed for all strontium-doped MSNs at concentrations higher than 500 μg/mL. No significant differences regarding the time of incubation in the hemolytic behavior of all MSNs was observed. A slight increase in the hemolytic activity was observed for MSi after 24 h of incubation.

## 3. Discussion

Multifunctional silica mesoporous NPs have been successfully synthesized by the sol–gel method for drug delivery systems, with optimum textural properties, such as high surface area, large pore volume, and uniform distribution of pore size [51]. However, as silica NPs lack apatite-mineralization activity, Ca doping could enhance their apatite-forming ability. The release of ions from ion-doped mesoporous glasses, such as Si and Ca, promote osteoblastic cells’ proliferation, which enhances the bonding to human bone [52]. Additionally, Mg, is a known trace element in the human body with a critical role in bone metabolism [22]. Hence, the synthesis of Mg-containing calcium silicate MSNs could be promising for use as bioactive mesoporous drug carriers [53]. A few recent studies report on the synthesis of MSNs containing strontium due to strontium’s anabolic and anticatabolic effect on bone metabolism, while Si and Sr can act synergistically on osteoporotic bone regeneration [18,21,54]. Thus, the combination of these properties from multiple ions incorporated in a single mesoporous nanoparticle should provide additional regenerative potential of MSNs apart from being solely carriers of reactive substances. In the present study, seven silica-based MSNs were successfully synthesized, using Ca, Mg and Sr as doping elements. All MSNs attained high surface areas, suggesting that the incorporation of ions such as Ca, Mg and Sr did not inhibit the formation of mesoporous structure and consequently their loading capacity. All MSNs presented a surface area highly above 350 m^2^/g, which has been considered appropriate for the adsorption of a range of molecules such as drugs and growth factors, and although the pore size was reduced compared to the pure silica (MSi), it was around 3 nm, which is considered acceptable for the effective loading and release of various drugs [5,55].

The FTIR spectra and XRD patterns of the different MSNs did not show remarkable differences. A slight difference is observed regarding the shoulder at 950–960 cm^−1^ which is more intensified in the spectra of MSi, MSiCa50 and MSiCa60 nanoparticles, indicating weak network connectivity, more porous structure and the formation of less bridging oxygens [20,36]. This finding was expected as mesoporous silica nanoparticles own an open network structure consisting of SiOx tetrahedrons, which enables the incorporation of alkali and/or alkali-earth cations. These cations, such as Ca^2+^, act as network modifiers that break the Si-O-Si bonds forming non-bridging oxygen groups (Si-O-NBO) and thus disrupt the glassy network [37]. According to Virgo et al. [56], different anionic structural units could be identified according to the number of NBO. A superscript number is assigned to the number of bridging oxygens (BO) present in silicate tetrahedral units, with Q0 representing zero BO and all NBO ([SiO_4_]^4−^), Q1 one BO and 3 NBO ([Si_2_O_7_]^6−^), Q2 two BO and two NBO ([SiO_3_]^2−^), Q3 three BO and one NBO ([Si_2_O_5_]^2−^) and Q4 four BO and zero NBO (SiO_2_) [38,57,58]. The addition of acidic oxides, as network formers, results in an increase in the number of BO. On the other hand, basic oxides act by disrupting the network, thus producing free oxygen ions (O^2−^) and NBO (O^−^) and reducing the number of BO (O^0^) [58], according to the Equation (1):

O^0^ + O^2−^ = 2O^−^ (1)

The kind and amount of Si-O structural units is related to the degree of depolymerization of the silicate network. Specifically, the degree of the polymerization should be decreased with basicity, confirmed by the decrease in the BO (bridging oxygens) [58]. In this respect, the addition of Ca seems to slightly affect the peak at 800 cm^−1^ which is attributed to the Si–O symmetric stretching vibration. Its decrease is even more pronounced after Mg and especially Sr addition. This decrease is associated with disrupted silicate network and verifies the incorporation of the ions into the glass network [19,36]. The incorporation of Mg seems to lead to stronger network connectivity and the formation of more bridging oxygens compared to Mg free samples, indicated by the decrease in the shoulder at 960 cm^−1^ [20]. However, the simultaneous broadening to lower wavenumbers of the peak at 1100 cm^−1^ has been associated with the stretching vibrations of the Si–O–2NBO in Q2 units, especially in cases of silicate networks with high modifier content. Thus, it is concluded that Mg behaves as network modifier in the MAk- and Sr-doped MSNs.

Although FTIR spectra and EDX analysis verify the incorporation of ions in the network, XRF analysis indicated that not all the nominal amounts of calcium, magnesium and strontium were incorporated into the silica network, a finding which is consistent with previous studies [36,59,60]. Keeping in mind that calcination above 450 °C is necessary for the efficient incorporation of Ca^2+^ and Mg^2+^ ions to the glass structure, all MSNs were calcined at 600 °C. Although higher calcination temperature could lead to higher incorporation, it could also cause further agglomeration of nanoparticles and a more dense structure jeopardizing the bioactivity of the MSNs by reducing the Si-OH content [61]. An interesting finding from XRF was that with the Sr incorporation, the amount of calcium was increased with the exception of the highest amount of Sr (MAkSr6). This may be attributed to the larger radius of strontium compared to that of Ca that creates more open silicate networks [62].

Bioactive glasses are highly reactive when they are soaked in human plasma [63]. Their dissolution results in the formation of a silica-based gel layer that is the first step towards the precipitation of a Ca-P layer on their surface [37]. Previous in-vitro studies revealed a relation between the percentage and kind of NBO and the development of silica-based and CaP layers. Ion exchange is favorable when Si-O-NBO bonds are abundant in the glass network. A minimum amount is needed for sufficient ion exchange that will enhance silica network dissolution and formation of silanol (Si-OH) groups, favoring the subsequent process of of SiO_2_ layer condensation and HCAp formation on the surface of the specimens. Taking into consideration the disrupted silica network resulted by the incorporation of all elements in the synthesized MSNs, all samples except MSi were capable of hydroxyapatite formation, as shown by FTIR and SEM-EDX analysis. Cauliflower-like apatite structure was formed on the surface of the Ca- and Mg-doped MSNs after 10 days immersion in SBF, as reported previously [64] for Ca-doped MCM-41 MSNs, while the Sr-co-doped MSNs presented only a roughening of their surface. This roughening may be associated with the growth of spherical granular precipitates, suggesting the onset of apatite formation, which could not be displayed on the respective SEM micrographs due to their significantly smaller size. However, the presence of crystalline Ca-P precipitates is verified by the double phosphate peak in the FTIR spectra of all MSNs after 10 days of immersion in SBF. However, the reduction in sharpness of this peak, can explain the absence of distinct cauliflower-like apatite structures on the SEM micrographs of the Sr-doped nanoparticles [65,66,67]. A synergistic effect of strontium and magnesium [68] may have also led to this result.

The interaction of nanoparticles with blood components such as platelets and RBCs can result in hemolysis, complement activation, inflammation, and thrombosis [69]. This cascade of events depends on the different properties of nanoparticles and consequently the evaluation of their hemolytic potential should be considered in relation to their overall toxicity. The tetra-alkyl ammonium groups on the membrane of RBCs can interact with the surface silanol groups of silica-based materials providing the main mechanism associated with their hemolytic properties. The hemolysis assay is commonly performed after 24 h of incubation, at body temperature (37 °C) and at different concentrations that range from 0.125 to 2.5 μg/mL. However it has been reported that the highest concentration that would be tested in relation to clinical intravenous administration should be 1 mg/mL [70]. The ASTM standard (ASTMF-756-08, 2009) defines non-hemolytic materials as those that present in vitro hemolysis up to 2%, while hemolysis percentages ranging from 2 to 5% are considered moderately hemolytic. Only materials that exert hemolysis beyond the 5% threshold are considered hemolytic. Nanomaterials presenting hemolysis >50% in vitro can cause immediate animal death after intravenous administration [70,71].

The hemolytic properties of various mesoporous nanoparticles have been recently evaluated. Many studies have shown hemolytic action of MSNs and generation of free radicals (ROS) [72] depending on concentration [73], porosity [74], size [75], geometry and curvature [72,74,76], surface area [76], surface silanol content [77,78], and surface charge [72]. Although MSNs exhibit lower hemolytic activity than nonporous silica nanoparticles [72,79], they still show high hemolytic potential which in many cases is above 50% [75,80]. Regarding the size, Lin et al. [75] reported a decrease with the increase in size from 25 to 225 nm, while Zhao et al. [76] found that MSNs with a diameter of 100 nm presented lower hemolytic activity compared to large MSNs (∼600 nm). The shape of MSNs has also been indicated as a contributing factor to hemolysis. Spherical nanoparticles have been considered as less hemolytic compared to their tubular counterparts, as have MSNs with a high aspect ratio (ratio of length over width) [72,81]. Although several factors have been considered important in monitoring the hemolytic behavior of MSNs with controversial findings in the literature, it seems that the most prominent one is the amount and density of silanols at the surface. As different synthesis methodologies have been employed, a variety of MSNs with varying properties and textural characteristics can be synthesized, and thus it becomes important to thoroughly evaluate their potential nanotoxicity before their therapeutic applications.

In the present study, MSNs presented low hemolytic activity related to the complexity of the system. At the concentration of 0.125 μg/mL only MSi, MSiCa50 and MSiCa60 can be considered hemolytic while MAk, MAkSr2, MAkSr4 and MAkSr6 present only moderate hemolytic activity even at concentrations of 1 mg/mL. It seems that the addition of magnesium and strontium prevents hemolysis, even though the size of the nanoparticles is smaller. From the ζ potential measurements it seems that no direct correlation exists with the hemolysis findings, although non-hemolytic MSNs presented less negative ζ potential compared to the hemolytic MSi nanoparticles. Ca^2+^ and other ions of modifying elements can be attached to silica matrices by a reaction with silanol groups on the surface [82], thus reducing their overall amount. A combination of z potential, particle size, agglomeration and pore volume may result in a smaller number of silanol groups available for cell contact [64], tailoring the total hemolytic activity. Despite a moderate hemolytic activity of doped MSNs when examined with plain RBCs, its complete elimination or significant reduction is anticipated as reported in other studies, due to the formation of a protective protein corona in the presence of the full human plasma [74]. Protein adsorption can modify the MSNs’ surface micro chemical environment, providing the actual biological identity of the synthesized MSNs. This is a subject of future investigation, which will clarify the exact role of each doping element on cellular viability and differentiation.

Periodontal connective tissue is rich in periodontal ligament and gingival fibroblast cells that are responsible for its regeneration. A lot of bioactive materials have been tested for their biocompatibility on human periodontal ligament fibroblasts (hPDLFs) and their ability to support their proliferation and differentiation. Ions released from bioactive glasses enhance periodontal ligament fibroblast osteocalcin expression and early mineralized tissue development [83]. A significant increase in cell proliferation was recorded after three days of incubation with hPDLFs for all the synthesized MSNs, suggesting their potential for application in periodontal tissue regeneration strategies. Despite their excellent biocompatibility and probable encouraging effect on hPDLFs differentiation, the success of periodontal regeneration is largely affected by the efficient control of local infections, caused by common periodontal pathogen growth [84,85]. In this respect, we evaluated the ability of the synthesized NPs to load and release moxifloxacin. Moxifloxacin was selected because of its excellent antibacterial efficacy and regenerative potential [86,87].

The loading extent is influenced by the loading procedure. Parameters such as type of solvent, time and temperature are important [88]. In vitro studies indicated that high efficacy depends on high release capacity. Moxifloxacin is a drug with a positive charge below pH 7.4 and negative above this pH value, so a strong electrostatic interaction could be developed between negatively charged MSNs and the positive molecules of moxifloxacin [89]. In order employ these electrostatic interactions, Li et al. [89] prepared two types of loading solutions with different pH levels. They confirmed that the applied loading solution with pH = 7.4 did not cause hydrolysis of the MSNs silica network, compared to strong base loading solutions and the higher moxifloxacin loading concentration was achieved with the negative charged inner mesopores. Similarly, due to these negative positive interactions, Lee et al. [90] confirmed that silica-based nanoparticles loaded with moxifloxacin are more effective in treating lethal pneumonic tularemia in a mouse model, compared to the respective amount of free drug. In the present study, the results obtained show that under the specific loading conditions, moxifloxacin could be efficiently loaded into most of the negatively charged silica-based MSNs. The highest loading combined with more sustained release was observed in MSiCa50 and MSiCa60 MSNs. Minerals belonging to the alkaline earth metal group (II), such as Ca and Mg, can bond with electrostatic bonds to carboxylic acid groups (–COOH) of moxifloxacin, affecting both loading and release kinetics [91]. The absence of these bonds could explain the lesser loading of MSi despite its high surface area. However, in the case of MAk MSNs, the addition of magnesium restricted drug loading compared to MSiCa50 and MSiCa60 MSNs, despite their similar textural characteristics. This may be attributed to the wide distribution of pore sizes evidenced by the steep desorption step (hysteresis loop) observed in BET analysis, and the absence of the typical hexagonal mesoporous order as verified by TEM. The incorporation of strontium slightly increased loading capacity compared to MAk only for MAkSr2 and MAkSr4 compositions, suggesting a dose-dependent effect. This was also accompanied by a more sustained release. The higher amounts of calcium in the glass of MAkSr2 and MAkSr4 MSNs available for electrostatic attraction to MOX carboxylic acid groups may explain the highest loading and slower release. A completely different profile was observed in the case of the MAkSr6 that presented extremely low loading efficiency. Possible explanations for this could be the low negative ζ potential of MAkSr6 nanoparticles, or a higher degree of aggregation due to their smaller size that may diminish the available silanols for bonding. As silanol groups and surface area can control the drug adsorption into the structure of MSNs and their loading capacity, the lowest surface area of these MSNs in combination with their smallest size and less negative charge may have accounted for the low drug uptake capacity. The MAkSr6 MSNs presented also large external porosity and interparticle voids that reduced the efficient MOX loading into the mesopores. Taking into account also their fast drug release, it can be assumed that moxifloxacin was mainly physisorbed on their surface rather being absorbed into their pores. A possible higher loading efficiency could be established with this type of MSNs if more stirring time was applied, to allow drugs to reach the inner pores. In the present study, a short time of stirring (2 h) was applied, and no optimization of loading conditions was investigated. Usually loading is performed my immersion of MSNs in an aqueous or organic solvent solution with the dissolved drug. Type of solvent, drug concentration and pH of the loading solution can affect the loading capacity either facilitating or impeding drug adsorption into the pores [92]. Although this is the most common method, a low cargo loading is usually observed in respect to the theoretical loading capacity [92]. By modifying the loading protocol in terms of solvent, concentration, temperature and stirring rate, higher loading can be achieved [93,94,95]. Increasing the time of soaking from 5 to 35 h resulted in a significant higher drug loading rate of SBA-15 mesoporous silica nanoparticles [93].

Great variations were recorded among the synthesized MSNs regarding MOX release. Such differences on the release rate and time, may be attributed to several factors, such as their surface area and pore size, water permeability, their particle size and size distribution, the complexity of MSN system, and drug loading levels. The overall release profile typically progresses slowly in three distinguished stages: an initial almost immediate and accelerated release, generally known as the “burst effect” (due to the surface bonded MOX), followed by a second slower and more controlled stage, and finally an extremely slow-going release where a plateau is almost recorded. Generally, most of the doped MSNs in the present study exhibited slow release rates. Interestingly, while the percentage of drug loading increased, the release rate decreased. According to Lai et al., a higher initial loading concentration can result in higher drugs absorption into the mesopores, minimizing dissolution rate and even eliminating supersaturation [96]. However, the unexpected high release rate recorded for the MAk MSNs may be attributed to its disordered mesoporous structure. High release in the first 24 h suggests a high amount of drug molecules on the external surface of MAk MSNs. In addition, variations in loading and release rates of all MSNs were recorded, that do not follow a specific trend. The only possible explanation for this may be the random incorporation process of slightly different CaO, SrO and MgO nanodomains into the siliceous mesostructure, depending on synergistic or antagonistic effects among the doping elements. Differences in degradation rates and subsequent pH variations, as well as external micro- or macro-porosity, inter-particle voids and defects on particle surfaces may accommodate the differences in drug loading/release kinetics that need further clarification in future studies. However, the biphasic release profile of most of the doped MSNs could provide a high concentration of moxifloxacin at first and then a slow release that could maintain the concentration of moxifloxacin at an optimized therapeutic level [88].

## 4. Materials and Methods

### 4.1. Synthesis of MSNs

The synthesis of silica-based MSNs, SiO_2_ (MSi), two types of SiO_2_CaO (50SiO_2_50CaO and 60SiO_2_40CaO mol, respectively) (MSiCa50 and MSiCa60) and SiO_2_CaOMgO with the nominal composition of akermanite (Ca_2_MgSi_2_O_7_) (MAk), was performed through a modified sol–gel method, using cetyltrimethylammonium bromide (CTAB) as agent for the mesoporous structure. Moreover, Sr-doped MSNs were successfully synthesized also with SrO being added in percentages of 2, 4 and 6% mol, replacing Mg in the nominal composition of akermanite (MAkSr2, MAkSr4 and MAkSr6, respectively). The reactants were sodium hydroxide (NaOH, alkaline medium), CTAB, tetraethyl orthosilicate (TEOS), Ca(NO_3_)_2_.4H_2_O, Mg(NO_3_)_2_.6H_2_O and Sr(NO_3_)_2_ from Sigma-Aldrich (now Merck KGaA, Darmstadt, Germany). The final molar ratios were 1TEOS/0.13CTAB/0.4NaOH/1280H_2_O. All the synthesized materials were dried at 60 °C overnight and underwent calcination at 600 °C for 5 h to remove CTAB.

### 4.2. Physico-Chemical Characterization

#### 4.2.1. Fourier Transform Infrared Spectroscopy (FTIR)

The characterization of the MSNs was performed by Fourier transform infrared spectroscopy (FTIR) with a Perkin Elmer Spectrometer (Perkin Elmer Inc., Waltham, MA, USA) in the transmittance mode (400–4000 cm^−1^), with a resolution of 2 cm^–1^ and 32 scans. For this purpose, KBr (Merck KGaA, Darmstadt, Germany) pellets were fabricated under 7 ton of pressure, with MSNs with a powder-to-KBr ratio of 1:100.

#### 4.2.2. X-ray Diffraction (XRD)

XRD patterns were obtained by a Rigaku Ultima diffractometer (Rigaku Corporation, Tokyo, Japan) with a Ni-filtered CuKa radiation source (λ = 0.1542 Å). The operating parameters were: 2θ scanning from 5° to 75°, step of 0.05° and contact time of 1 s.

#### 4.2.3. Particle Size and Z-Potential Measurements by Laser Dynamic Light Scattering (DLS)

The particle size measurements were performed by a dynamic light scattering (DLS) analyzer (Zetasizer Nano, Malvern Instruments, Nano ZS, ZEN 3600, Malvern, UK) equipped with a 532 nm laser, using angle measurements of exactly 90° at 25 °C. The samples were measured in suspension form, using aqueous solution of NaCl (10^−4^ M) after sonication at 25 °C. For all samples, experiments were performed in triplicate and ζ-potential value corresponds to their mean average.

#### 4.2.4. Brunauer–Emmett–Teller (BET) and Brunauer–Joyner–Halenda (BJH)

The mesoporous structure of the MSNs was determined by N2 adsorption/desorption at −196 °C (Autosorb-1MP, Quantachrome Instruments, Boynton Beach, FL, USA), using the Brunauer–Emmett–Teller (BET) and Brunauer–Joyner–Halenda (BJH) methods.

#### 4.2.5. Scanning Electron Microscopy (SEM) with Energy Dispersive X-ray Analysis (EDX)

The morphology of nanoparticles was assessed by SEM (Auriga Base, Carl-Zeiss, Oberkochen, Germany) while associated EDX was performed in order to qualitatively investigate the sample compositions.

#### 4.2.6. Transmission Electron Microscopy (TEM) Analysis

For TEM imaging, the NPs samples were dispersed in an ethanol solution and were submitted to sonication for 10 min. Then, a drop of the suspension was placed onto a Lacey Carbon Film (Agar Scientific Ltd., Essex, UK). For imaging and morphology analysis, a latest generation Field Emission Gun Transmission Electron Microscope (Talos F200X) was utilized.

#### 4.2.7. X-ray Fluorescence Spectroscopy (XRF)

Bulk analysis of the specimens was determined by a Bruker S4-Pioneer XRF wavelength dispersive spectrometer equipped with an Rh tube, with five analyzing crystals: LIF200, LIF220, LIF420, XS-55 and PET. The detectors were a scintillation detector or a gas-flow proportional counter, or a combination of the two. Samples were analyzed at 60 kV and 45 mA tube-operating conditions. Specimens were prepared as glass beads by the fusion process using lithium tetraborate (LiT or Li_2_B_4_O_7_) as a flux. The ratio of specimen/flux was 1/8. The mixture was fused in a platinum crucible in a Vulcan fusion machine (Fluxana, Bedburg-Hau, Germany).

#### 4.2.8. Apatite Forming Ability in c-SBF

The MSNs were immersed in c-SBF solution at a concentration of 1.5 mg/mL [97,98] and were maintained in an incubator (Incucell 55, BMT Medical Technology, Zábrdovice, Czech Republic ) at 37 °C. SBF was replaced at 6 h and 24 h after initial immersion and then after every 48 h. The samples were collected after 10 days of immersion in SBF and left to dry at room temperature before SEM and FTIR analyses.

#### 4.2.9. Evaluation of Drug Loading

The determination of MOX (Pharmathen SA, Athens, Greece) loading was performed using the indirect method (measuring the amount of the drug that was not loaded into the nanoparticles). For that, 100 mg of the mesoporous nanoparticles was dispersed in 10 mL of MOX methanol solution (10 mg/mL) and vigorously stirred for 2 h at 37 °C. The MOX-loaded nanoparticles were separated from the suspension by centrifugation at 5000× *g* for 15 min, and were then dried at room temperature. Each supernatant, after proper dilution, was analyzed using HPLC through a Shimadzu HPLC system (model LC-20AD, Tokyo, Japan). An Athena C18 (CNW Technologies, Düsseldorf, Germany) 5 μm, 120 Å, 250 mm × 4.6 mm analytical column was used, with the flow rate set at 1.0 mL/min and temperature at 25 °C. The mobile phase was prepared by mixing methanol and water (pH adjusted to 2.57 using 85 wt% orthophosphoric acid and triethylamine solution) in the ratio 55:45. The wavelength of UV detection was set at 293 nm and the injection volume at 10 μL, whereas the quantification of MOX was based on a calibration curve previously prepared at 0.025, 0.05, 0.1, 0.5, 1, 2.5, 5, 30, 40 and 50 μg/mL MOX to mobile phase. The drug loading (DL) was calculated using the following equation:

DL (%) = [weight of drug in nanoparticles]/[weight of nanoparticles] × 100(2)

#### 4.2.10. In Vitro Drug Release Studies

In vitro drug release studies were performed in a Dissolution Apparatus (Distek, Evolution 2100C, North Brunswick Township, NJ, USA), provided with an autosampler (DS Evolution 4300) using the basket method (USP I method). Drug-loaded MSNs enclosed in a dialysis cellulose membrane bag with a molecular weight cut-off of 12 400, were placed onto appropriate sample holders. The dissolution medium was 250 mL of SBF (pH = 7.4, 37.0 ± 0.5 °C) and the stirring rate was kept constant at 50 rpm. The HPLC method was used to assess the moxifloxacin content. In particular, at predetermined time intervals, 2 mL of the release medium were removed, filtered and assayed, while the experiments were performed in triplicate for each MSNs group.

### 4.3. Biological Properties Evaluation

#### 4.3.1. In Vitro Biocompatibility Assay

Primary cultures of human periodontal ligament fibroblasts (hPDLFs) were established from a periodontal ligament tissue biopsy of a healthy donor during the surgical extraction of a third molar. The protocol for the establishment of fibroblasts’ culture was approved by the Ethical Committee of the Dentistry Department at the Aristotle University of Thessaloniki, Greece (#35/07-05-2018). Specifically, the soft tissue was minced and digested in a solution of collagenase type I in concentration of 3 mg/mL and 4 mg/mL dispase for 1 h at 37 °C. Then, soft tissue segments were placed in 25 cm^2^ culture flasks and cells were expanded in DMEM culture medium (Dulbecco modified Eagle medium- Biosera, Nuaille, France) supplemented with 10% FBS serum (Fetal bovine serum 10%, Gibco-BRL, Thermo Fisher Scientific Inc., Waltham, Massachusetts, USA) and antibiotics/antimycotics (penicillin, amphotericin B, streptomycin (Gibco-BRL, Thermo Fisher Scientific Inc., Waltham, MA, USA)). The flask was placed in a sterilized incubator at 37 ± 1 °C and 5% CO_2_, 95% atmospheric pressure, 100% humidity. When the flask became confluent, cells were transported by trypsinization (solution trypsin 0.25% 1 mM EDTA solution (GIBCO, Invitrogen, Thermo Fisher Scientific Inc., Waltham, MA, USA) to a larger flask 75 cm^2^ (passage 1). Cells from passage 4 to 5 were used in this in vitro study.

For biocompatibility assay, the MSNs were disinfected with UV light for 90 min. MSNs suspensions (stock = 1 mg/mL) weighted with electronic precision scale (OHAUS Europe GmbH, Im, Greifensee, Switzerland) and their dilutions were prepared with cell culture medium (DMEM). Eluates of the tested materials were prepared by incubating the MSNs with culture medium at three concentrations (60 μg/mL, 125 μg/mL and 250 μg/mL) for 24 h. MTT (3-(4,5-dimethylthiazol–2-yl)-2,5-diphenyltetrazolium bromide) assay was used to evaluate the potential cytotoxicity of tested MSNs on hPDLFs. Overall, 3 × 104 cells/well were placed in 96-well plates and left to attach for 24 h. Then, 200 μL of culture medium was removed from each well and replaced with the same amount of the respective eluate. Cells with the eluates were left at 37 °C in humidified 5% CO_2_ atmosphere for 24 and 72 h. Evaluation of cell proliferation was calculated through the mitochondrial dehydrogenase activity of living cells which was verified by measuring the optical density of the solutions after the transformation of the yellow tetrazolium salt into blue formazan crystals and their subsequent dissolution with dimethyl sulfoxide (DMSO, Merck KGaA, Darmstadt, Germany). Optical density was determined by spectrophotometry at a wavelength of 545 nm and a reference filter of 630 nm by a microplate reader (Epock, Biotek, Inc., Winooski, VT, USA). All results were calculated as an average percentage of the control (cells seeded with culture medium without eluates). Statistical analysis of MTT assay results was performed with SPSS. The level of statistical significance was set at 0.05.

#### 4.3.2. Hemocompatibility Assay

For the hemocompatibility assay, whole blood was obtained from healthy adults after written consent, from the French Blood Establishment (Etablissement Français du Sang, EFS, Toulouse, France), responsible for ethics statements. After 5 min of centrifugation at 1200 rpm, red blood cells (RBCs) were separated from plasma and leukocytes and were washed three times with phosphate buffer saline (PBS). Red blood cells were then mixed separately with different concentrations of MSNs (12.5, 30, 60, 125, 500 μg/mL) from a stock solution (10 mg/mL) for 60 min and 24 h of incubation at 37 °C (Thermomixer-Biosan, Riga, Latvia) by gentle inversion in the tube as previously described [33,72,99]. In detail, RBCs were diluted in PBS in a final suspension consisted of 5% volume erythrocyte (final volume: 1 mL) (hematocrit: 5%).

The supernatant of untreated RBCs was used as negative control (Ctrl-) and that of RBCs treated with lysis buffer was used as positive control. Following centrifugation at 1000 rpm for 1 min, the supernatants of treated RBCs after the predetermined incubation times was collected and placed in a microplate reader (Thermo Fisher Scientific Inc., Waltham, MA, USA) to measure the absorbance of the released hemoglobin, at 541 nm with reference wavelength at 700 nm. The percent of hemolysis was calculated from six independent experiments done in triplicates, according the following Equation (2):

Hemolysis % = [sample absorbance-negative control]/[positive control-negative control] × 100(3)

## 5. Conclusions

In this study, seven silica-based MSNs doped with Ca, Mg and Sr were successfully synthesized. All doped MSNs revealed the formation of hydroxycarbonate apatite on their surface after 10 days of immersion. The increased percentage of Sr doping led to a reduction in particle size, without significantly affecting the textural characteristics of the nanoparticles. The addition of Mg and Sr up to specific concentrations improved the hemolytic activity and cell proliferation, while maintaining sufficient moxifloxacin loading and sustained release rates. The physicochemical and biological properties of these doped MSNs make them excellent candidates for hard tissue regeneration as functional fillers in polymeric composites, scaffolds, coatings for metallic implants or injectable bioactive cements. Further tailoring of the synthesis process and drug loading conditions could provide Ca-, Mg- and Sr- co-doped MSNs with excellent properties that may combine optimum textural characteristics with the capacity to increase cell proliferation and differentiation in the osteoblastic lineage of periodontal ligament cells for periodontal tissue regeneration.

## Figures and Tables

**Table 1:** Textural properties of the synthesized mesoporous silica-based nanoparticles (MSNs).

Silica-Based Mesoporous Nanoparticles (MSNs), mol%						
Composition	Surface Area (m^2^/g) S_BET_	Pore Volume (cm^3^/g)	Average Pore Size (nm)	Size (nm)	ζ-Potential	PDI
MSi	1279	1.003	2.4	408.8	−26.700	0.362
MSiCa50	726	0.753	2.7	437.9	−22.100	0.436
MSiCa60	741	0.805	2.7	534.7	−19.300	0.507
MAk	757	0.989	3.0	522.9	−20.800	0.426
MAkSr2	700	1.618	3.0	304.9	−21.700	0.674
MAkSr4	737	1.994	3.0	207.4	−16.000	0.699
MAkSr6	668	1.812	3.1	151.9	−15.700	0.708

**Table 2:** Chemical composition of the synthesized MSNs as detected by XRF in mol%.

Sample	SiO_2_		CaO		MgO		SrO		Total
	N ^1^	XRF	N^1^	XRF	N ^1^	XRF	N^1^	XRF	
MSi	100	100.00	-	-	-	-	-	-	100
MSiCa50	50	81.57	50	18.43	-	-	-	-	100
MSiCa60	60	80.69	40	19.31	-	-	-	-	100
MAk	40	66.12	40	18.41	20	15.46	-	-	100
MAkSr2	40	58.74	40	27.04	18	13.19	2	1.03	100
MAkSr4	40	64.45	40	23.34	16	10.46	4	1.74	100
MAkSr6	40	72.93	40	15.95	14	8.70	6	2.42	100

^1^ N = Nominal composition.

**Table 3:** Moxifloxacin drug loading (%) into the MSNs.

Sample	% MOX Loading in the MSNs
MSi	15 ± 2
MSiCa50	38 ± 1
MSiCa60	21 ± 1
MAk	11 ± 3
MAkSr2	12 ± 1
MAkSr4	14 ± 1
MAkSr6	2 ± 0.1

## Data Availability

The data presented in this study are available on request from the corresponding author. The data are not publicly available due to privacy issues.

## Abbreviations

MSNs	Mesoporous Silica-based Νanoparticles
XRD	X-ray Diffraction
SEM	Scanning Electron Microscopy
EDX	Energy Dispersive X-ray Analysis
TEM	Transmission Electron Microscopy
FTIR	Fourier Transform Infrared Spectroscopy
XRF	X-ray Fluorescence Spectroscopy
BET/BJH	Brunauer Emmett Teller and Brunauer Joyner Halenda
DLS	Dynamic Light Scattering
HPLC	High Performance Liquid Chromatography
BG	Bioactive Glasses
MBGs	Mesoporous bioactive glasses
MOX	Moxifloxacin
NPs	Nanoparticles
MTT	3-(4,5-dimethylthiazol–2-yl)-2,5-diphenyltetrazolium bromide
RBCs	Red Blood Cells
BO	Bridging Oxygens
NBO	Non Bridging Oxygens
HCAp	Hydroxycarbonate Apatite
hPDLFs	Human Periodontal Ligament Fibroblasts
CTAB	Cetyltrimethylammonium Bromide
TEOS	Tetraethyl Orthosilicate
DL	Drug Loading
SBF	Simulated Body Fluid
DMEM	Dulbecco’s Modified Eagle’s Medium
DMSO	Dimethyl Sulfoxide
